# The Impact of Pain Education Interventions for Cancer Survivors and Caregivers: A Systematic Review with Meta-Analysis

**DOI:** 10.3390/cancers16132468

**Published:** 2024-07-05

**Authors:** Sofía Hernández-Hernández, Alejandro Heredia-Ciuró, Javier Martín-Núñez, Andrés Calvache-Mateo, Alba Navas-Otero, Laura López-López, Marie Carmen Valenza

**Affiliations:** Department of Physiotherapy, Faculty of Health Sciences, University of Granada, Av. De La Ilustración 60, 18016 Granada, Spain; shernandez@correo.ugr.es (S.H.-H.); ahc@ugr.es (A.H.-C.); javimn@ugr.es (J.M.-N.); andrescalvache@ugr.es (A.C.-M.); e.albanavas@go.ugr.es (A.N.-O.); lauralopez@ugr.es (L.L.-L.)

**Keywords:** pain education, cancer survivor, caregiver

## Abstract

**Simple Summary:**

Implementing educational programs for patients and their caregivers has been suggested as an effective method to help alleviate pain associated with cancer. The purpose of this study was to compile and review the current pain education interventions for cancer patients and their caregivers using a standardized methodological approach, and to evaluate the impact of these interventions on pain. These findings are significant for healthcare professionals as they provide a foundation for motivating cancer patients to engage actively in their treatment. Implementing these results could save time and resources for healthcare providers, thereby enhancing the quality of treatments.

**Abstract:**

Introduction: Cancer-related pain is a global health-related problem associated with functional impairment, anxiety, depression, and reduced quality of life. The use of educational interventions for patients and their caregivers has been proposed as a promising tool for overcoming pain in cancer. The aim of this study was to summarize by means of a standardized methodological systematic revision the actual pain education intervention used in cancer patients and their caregivers and to analyze its effects on pain. Methods: A search was conducted through PubMed, Web of Science, Scopus and Cinhal from their inception to September 2022. Randomized controlled trials which included pain education interventions were identified. Two reviewers performed independent data extraction and methodologic quality assessments of these studies. Results: A total of seven studies was included in the study. The meta-analysis showed that pain education interventions have a significant effect on the worst pain; however, there was no effect on average pain. Conclusions: Pain education interventions addressed to patients and their caregivers could have positive effects on cancer-related pain. It is recommended that a minimum of three sessions of about one hour’s duration be held once a week. Further research needs to be carried out and analyzed on the effects over the long term. Pain education interventions show positive results in improving pain in cancer patients regardless of etiology or extent of the cancer. Studies with better methodological quality should be carried out to address specific components related to education interventions.

## 1. Introduction

Globally, cancer incidence is increasing at a similar rate to life expectancy due to screening methods and better treatments, and there will be an estimated 21.4 million cancer patients by 2030 [1,2]. As survival rates improve, new challenges emerge related to the impact of living with the long-term effects of cancer treatment.

Symptoms and emerging health problems, often secondary to the cancer, are experienced by cancer survivors for years following primary cancer treatment [3]. More than one-third of the cancer patients report moderate or severe pain present in all stages of cancer, rated from 50% at the time of diagnosis to 50–60% during treatment [4]. Therapeutic approaches to cancer-related pain remain complex due to its heterogeneous etiology and the varied cancer treatments [5]. Despite the existence of guidelines for pain management in cancer, reports show pain is still a health-related problem associated with functional impairment, anxiety, depression, and reduced quality of life [6].

Inadequate pain management seems to be caused by professional-related as well as patient-related barriers. Furthermore, patients often impede their own treatment due to lack of knowledge about analgesics and their side effects, fear of drug tolerance and drug addiction, nonadherence to treatment regimens, and poor communication about their concerns about pain to healthcare providers [7]. To address these problems, greater emphasis needs to be placed on educating patients and stimulating them to participate actively in their own pain treatment program [8]. When considering the increasing recognition of informal caregivers’ role in managing patients’ pain, surprisingly there is a lack of clear methods and recommendations on how to prepare caregivers for managing patients’ pain at home. Additionally, both patients and their usual caregivers are required to make numerous judgments and choices daily about how to achieve optimal pain control, but they are not routinely included together in pain educational programs [9].

Educational interventions are proposed as a promising tool for overcoming pain. A pain education intervention was defined by Bennett et al. as “an intervention that results in improved knowledge, attitudes or both, which in turn alters the behaviour of the patient or professional and results in better pain outcomes” [10]. It was recommended by the guidelines of the Agency for Heathy Care Policy and Research as a method to improve the control of cancer pain, along with modifying knowledge and beliefs of the patients [11].

In this line, the effectiveness of educating patients and their caregivers on cancer pain management has shown some positive effects in different studies. However, these types of interventions can be complex, and require consideration of content, timing, frequency, duration, and format. The aim of this study was to summarize by means of a standardized methodological systematic revision the actual pain education intervention used for cancer patients and their caregivers and to analyze its effects on pain.

## 2. Materials and Methods

### 2.1. Search Strategy and Eligibility Criteria

This systematic review was conducted and followed the guidelines of the Preferred Reporting Items for Systematic Reviews and Meta-Analyses (PRISMA) statement and the Cochrane Collaboration guidelines for reviewing interventions [12,13]. The protocol of this systematic review has been registered on PROSPERO 2022 (registration number CRD42022309460).

We carried out a wide search of the literature for articles indexed on PubMed, Web of Science, Scopus, and Cinhal of randomized controlled trials databases from their inception to September 2022. We carried out a search strategy in MEDLINE using the following steps: (1) development of key words by examining relevant key terms used in existing systematic reviews, (2) a thorough examination of the MeSH Database in reference to the terms “cancer”, “patient education”, “therapeutic education”, “medical oncology”, and “nursing oncology”, (3) and expert guidance and review by a specialist. This search strategy was tested and refined to confirm it was the most effective strategy for this review. Then, this strategy was adapted to index across other databases.

We screened the references of relevant reviews for additional studies that could be potentially included in this review. Non-English language studies, when a translation could be made available, were considered for inclusion too.

We applied the PICOS [13] (participants, interventions, comparisons, outcome, and study design) model to define the research question. The inclusion criteria were as follows: (1) cancer survivors, at any stage of their illness, receiving any type of medical treatment; (2) education in cancer interventions including caregivers; (3) the education intervention had to be compared to a control intervention or no treatment; (4) cancer-related pain was included in the outcomes; (5) only randomized clinical trials and pilot randomized clinical trials were included.

The inclusion criteria for considered as an education intervention were based on the definition proposed by Bennett et al. [10], which includes interventions where there is an improvement in knowledge, attitudes, or both, of pain management.

### 2.2. Study Selection and Data Extraction

To reduce the selection bias potential, two authors (SH; JM) independently performed the literature search and disagreements were resolved by further consultation with a third author (CV). The search process included removing duplicates and screening titles, abstracts, and eligible full texts.

The search was carried out by two authors (SH; AH), independently to minimize potential selection bias. They each conducted the literature search, removed duplicates, and screened titles and abstracts. Any disagreements were resolved through consultation with a third author. Similarly, data extraction and quality assessment were also carried out independently by two authors.

### 2.3. Assessment Quality and Risk of Bias in Included Studies

We used the Downs and Black quality assessment method [14] to assess the methodological quality of the included studies. It includes 27 items comprising five subscales (study quality, external validity, study bias, confounding and selection bias, and study power). Quality assessment is classified as follows: excellent when it reaches a score of 26 points or more, good between 20 and 25, fair between 15 and 19, and poor when it is less or equal to 14 [15]. This scale has been ranked as one of the six highest-quality assessment scales suitable for use in systematic reviews due to its high validity and reliability.

We assessed the risk of bias using the Cochrane Risk of Bias Tool for randomized controlled trials method [16]. It includes 7 elements with 6 subscales (selection bias, performance bias, detection bias, attrition bias, reporting bias, and other bias). A study is considered to have high quality when there is low risk for each domain. Quality is considered fair when one criterion is not met (i.e., high risk of bias for one domain) or two criteria are unclear, and there is no known important limitation that could invalidate the results. Quality is considered poor when one criterion is not met or two criteria are unclear, and there are important limitations that could invalidate the results; and when two or more criteria are listed as having high or unclear risk of bias.

The GRADE system (Grading of Recommendations, Assessment, Development, and Evaluation) facilitated the qualitative analysis and classification based on the evidence levels of the studies included in our review. This system evaluates five domains: study design, imprecision, indirectness, inconsistency, and publication bias [17,18].

The GRADE criteria were utilized to assess these five domains. In the domain of study design, recommendations could be downgraded by one level if there was uncertainty or a significant risk of bias, accompanied by notable limitations in the effect estimate. For inconsistency, recommendations could be downgraded by one level if point estimates varied significantly between studies, confidence intervals had minimal overlap, or the I2 statistics indicated considerable or substantial heterogeneity. In the case of indirectness, recommendations could be downgraded if there were significant variations in interventions, study populations, or outcomes. For imprecision, recommendations could be downgraded by one level if there were fewer than 400 participants for continuous data [19].

### 2.4. Meta-Analysis

We used the Review Manager 5 (RevMan 5) software to perform a meta-analysis on all studies that presented pain values. When data were insufficient for meta-analyses purposes (e.g., no means provided, no standard deviation provided), we contacted trial authors if it was possible.

When *p*-values or 95% confidence intervals were given and standard deviations were missing, these were calculated via the embedded Review Manager calculator. We used the Q statistic and I2 to examine statistical heterogeneity. Visual inspection of the forest plots for outlier studies was also undertaken. I2 describes the percentage of total variation across studies that is due to heterogeneity rather than chance. We interpreted I2 of more than 50–90% as an indicator of substantial heterogeneity. When homogeneity was observed, a fixed effects model was used and expressed effects as standardized mean differences (SMD) with accompanying confidence intervals.

## 3. Results

**Figure 1:**
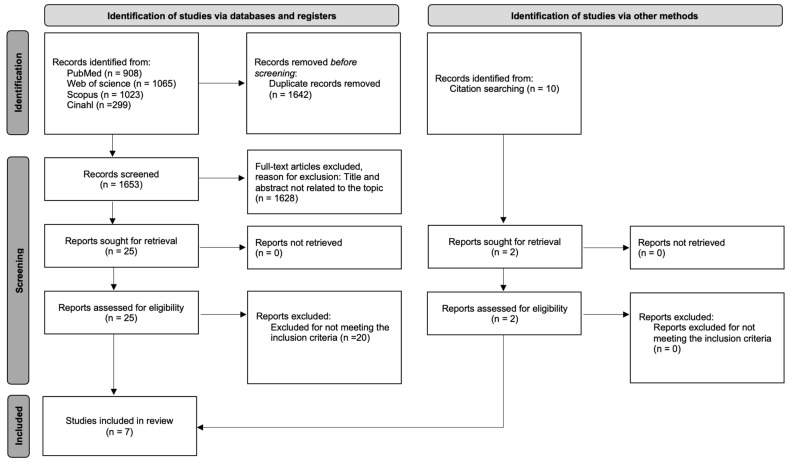
PRISMA flowchart.

A flowchart showing the process of the research, screening, and selection of the studies is presented in Figure 1. A total of 3295 studies was obtained from the database searches. After removing all duplicates, 1628 studies were obtained. A screening according to title and abstract was performed, obtaining a total of 25 articles. Ten articles were excluded because they did not meet the necessary inclusion criteria.

The reference lists of relevant studies was checked to see if the references included reports of other studies that might be eligible for the review. No other studies were identified by this method. Finally, seven studies were included in the review. Five of them were included in the meta-analysis corresponding to average pain and six were included in the meta-analysis corresponding to worst pain.

This review included six RCTs [20,21,22,23,24,25] and one pilot RCT [26]. All studies conducted a comparison between an intervention group (education program) and a control group (usual care) [20,21,22,23,24,25,26]. The characteristics of the included studies are shown in Table 1. A total of 946 cancer survivors was included in the reviewed studies, with the mean age of the participants ranging between 53.3 ± 10.2 and 64.32 ± 11.4 years in the intervention group and 55 ± 14.38 and 67.38 ± 11.4 years in the experimental group.

Four studies recruited various types of cancers [20,22,25,26], one study recruited patients with head and neck cancer [21], and two studies did not report the type of cancers they studied [23,24]. Extent of disease (local, regional, metastatic) [20,21] was reported only in two studies; four studies selected only patients in the metastatic stage [22,23,24,25] and one study did not report the disease [26]. Treatment types described as the received treatment aimed to resolve cancer in the reviewed studies were chemotherapy, radiotherapy, surgery, hormonal therapy, and biotherapy. The percentage of patients receiving each type of treatment was reported in six studies excluding the one conducted by Koller et al. [26]. The most common treatments received were chemotherapy, radiotherapy, and hormone therapy [4,20,22,25].

All of the included studies reported patients had informal caregivers [26]. Most of the studies did not report what relationship the patient had with the informal caregivers. Only two trials reported if they were spouses, children, or siblings [21,23]. Keefe et al. and Lin et al. reported sex and age [23,24], which ranged from 38.2% to 39.34% male and from 46 ± 12.59 to 58.48 years.

When the Cochrane Risk of Bias Assessment was applied, four studies presented high risk of bias [20,21,24,25], two studies presented some concern of bias [22,25], and only one study presented low risk of bias [23].

We applied the GRADE recommendations to evaluate the level of evidence for the use of pain education interventions for cancer survivors and caregivers and obtained low recommendation for average and worst pain. The decrease in the assessment of certainty was primarily due to the high risk of bias.

To facilitate the understanding of the pain education interventions due to heterogeneity, components used in each intervention were classified according to taxonomy proposed by Hors-Fraile et al. [27]. Based on this classification, the component of pain education most used in the experimental intervention was “pain knowledge”, “advising medication use”, “barriers for pain management”, and “advising changing routines”. The interventions used in the studies were based on enhancing or improving pain knowledge by giving pain education to patients and caregivers. Similar components were identified in the pain education interventions: how to register pain was explained to patients and their caregivers in three studies [20,21,24], help-seeking behavior was a component of another two studies, enhancing communication was applied in two studies, medication use also in two studies, teaching coping skill in three studies, and finally, coaching was used in another two studies. A detailed description of the used interventions can be found in Table 2.

The dose of pain education interventions was heterogeneous: the duration of intervention ranged from 1 to 6 weeks, the frequency of intervention varied between every 3 days and biweekly, and the intervention time ranged from 30 to 60 min. Most of the interventions provided complementary materials, such as a pain brochure, audiocassette, or visual media, to be able to review the information explained in the sessions at home.

The majority of educational materials delivered to patients and caregivers was booklets and videotapes. The study of De Wit et al. [20] implemented a pain diary that had to be filled in by patients and caregivers.

The form of application of the intervention was mainly mixed, with one first presential session, and a following telephone session to deepen education or to solve patients and caregivers’ doubts. All interventions were supervised and individual.

Pain-related outcomes were the most common outcomes explored in these studies. Pain experience was evaluated in six studies [20,21,23,24,25,26] and pain intensity in two studies [20,22]. The assessment tools are described in Table 2.

After treatment intervention, three studies showed improvements in pain experience outcomes in the experimental group concerning baseline and compared to the control group [20,24,26]. In terms of pain intensity outcomes, two studies concluded that there was an improvement in the experimental group concerning baseline compared to the control group [20,22].

**Figure 2:**
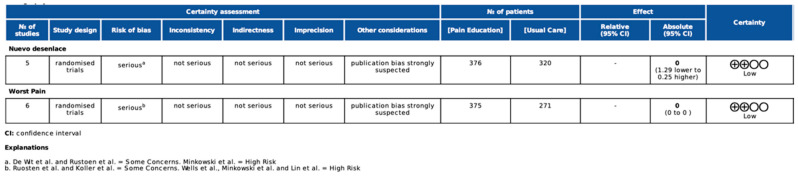
Grade system [20,25,26].

The assessment of the quality of the evidence using GRADE is fully described in Figure 2.

### Results Obtained in Meta-Analysis

**Figure 3:**
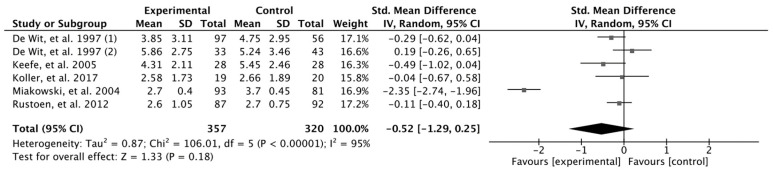
Average pain meta-analysis [20,22,23,25,26].

**Figure 4:**
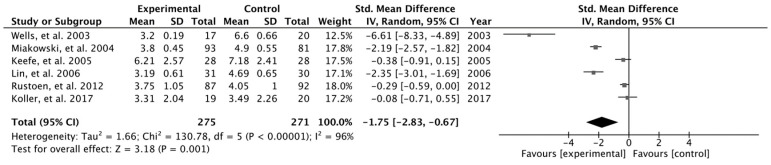
Worst pain meta-analysis [21,22,23,24,25,26].

The results obtained from the meta-analysis of average pain and worst pain are presented in Figure 3 and Figure 4, respectively. According to the results related to average pain, the pooled mean difference (MD) did not show a significant overall effect of pain education intervention compared to that of the control group (MD = −0.52, 95%, CI = −1.27, *p* = 0.173). The results showed heterogeneity, detecting significant variability of I2 = 95%. For worst pain, the pooled mean difference (MD) showed a significant overall effect of the pain education intervention: the experimental group compared with the control group (MD = −1.9, 95%, CI = −3.77, *p* = 0.046). The results showed heterogeneity, detecting significant variability of I2 = 98%.

## 4. Discussion

The present review aimed to summarize and present the actual efficacy of pain education interventions on cancer-related pain for patients and their caregivers. Our findings show that there is limited but optimistic evidence demonstrating that pain education is useful for pain management in cancer patients. The main results of this review were the beneficial effects of pain education interventions on pain experience and pain intensity, especially in average pain and worst pain intensity reduction. In this sense, these results have the potential to reduce the public health and healthcare impact of cancer pain, as has been also observed in other populations [28].

The variability of the included studies in terms of differences in the sample distribution according to the type of cancer, treatment received, or clinical stage may impact our conclusions. Other reviews [10,29,30] of pain education in cancer patients have yet reported the heterogeneity of the populations included.

In general, pain education programs provide an individualized, inexpensive, and easy source of information to improve patients and their caregivers’ pain knowledge and beliefs [31]. These programs are targeted towards the needs of the patient and his/her caregivers, and therefore may provide tailored educational material fitting the specific requirements of the patients. This individualization is a plus point for those interventions, as it allows their application regardless of the age, the stage of treatment the patient is at, or the clinical cancer profile. This is supported by studies such as the systematic review carried out by Champarnaud et al., who analyzed the effectiveness of patient education interventions in older adults with cancer, obtaining positive results on pain, patient knowledge, anxiety, and depression [32]. Other RCTs have shown reduction in cancer-related pain in preoperative cancer settings [33], after undergoing breast surgery [34] or during chemotherapy treatment, also improving the quality of life of cancer patients [35]. In this line, the implementation of pain education programs in the management of cancer pain could show benefits for the quality of life of cancer patients, as observed in other patients with chronic pain [36].

By reviewing the detailed evidence from seven RCTs, considerable insight has been gained into the components and mechanism which underpin educational interventions to improve cancer pain management. Although the components of the intervention were heterogeneous, several articles were based on the article by De Wit et al. in 1997 [20]. Based on this information, it has been possible to suggest the key components of educational interventions for cancer pain, including the importance of identifying, through an individual interview, the needs, and deficiencies of each individual and his or her caregiver before intervention. After that, specific information should be given about the cancer, on how to manage breakthrough pain (by means of relaxation, massage, exercise), and how and why the prescribed medications should be taken. It is also important to work on the patient’s social environment, by helping them to adopt seeking behaviors and teaching coping skills. This would help them to face their fears and improve their communication both with caregivers and health professionals. This process would be carried out based on the information given by healthcare professionals [37], making it important to always provide material (brochures, books, audiotapes) with the session’s information, so that it can be reviewed whenever needed.

This review suggests that the minimum number of education sessions should be three sessions, once a week and with a duration per session of no more than one hour. In each session, it is recommended to guarantee time for questions and clarification of concepts. A recent guideline for clinicians developed by Nijs et al. supports these findings. They recommend three educational sessions of 30 min, including the principal caregiver [38].

As Prevost et al. [39] describes, for pain education to be effective, there must be involvement on the part of both the patient and the caregiver. This implies a change in the role that patients and their families have had throughout history, being passive spectators of their illness. This paradigm shift is not easy for many of them, leading, in many cases, to a failure of educational therapies. This stands out in some of the RCTs presented in this review, in which no significant differences were found between the intervention group and the control group. It is proposed, for future research, to study which determinants make patients most sensitive to role change so an effective education approach can be made.

### Limitations

The articles included in the review had slight differences in patient cancer profiles and assessed a variety of heterogeneous interventions. There is no actual consensus in the literature about the accepted taxonomy to define pain education, number of sessions or intervention design. Some trials apply interventions without a previous detection of individual requirements, based only on a biomedical model, and do not consider the psycho-social spheres.

Future studies should include some factors that could modify the effectiveness of pain education, such as the type of pain, or the cancer stage. In addition, the included studies did not consider variables related to psycho-emotional status. It could be interesting to include them in future studies because, in other pathologies, pain education interventions have been effective in anxiety [40]. Moreover, the role of caregivers in the pain education intervention should be better specified in future studies.

The studies included in our review presented a low quality, with some risk of bias in some of them. Few of them presented groups with a small sample size. Although efforts were made to minimize bias and heterogeneity, these differences may have contributed to the variability in the results. All this means that the results’ interpretation and the conclusions drawn in this review should be taken with caution for clinical application.

For these reasons, this review highlights the need for further research in the field of higher quality, with defined interventions, dose, and frequency, and long-term follow-up of outcomes to see if the benefits observed in this review last over time, providing a solid basis for the implementation in clinical practice.

## 5. Conclusions

In conclusion, pain education programs in cancer patients showed significant improvement in pain. However, this review cannot support long-term effects since we were able only to meta-analyze post-treatment outcomes. Due to its high heterogeneity, it has been difficult to summarize the main characteristics of pain education programs, although it is suggested that they can be used in any type of cancer and its stages. It is recommended that a minimum of three sessions of about one hour’s duration be held once a week. Further research needs to be carried out and analyzed on the effects over the long term.

Despite these limitations, pain education programs present a promising option for improving the care and management of cancer patients. This paper is the first review to provide an overview of the current state of the literature on pain education programs in cancer patients. This study shows a range of different pain education interventions and their compositions, including different resources. These results are important for healthcare professionals since they establish a starting point for encouraging cancer patients to take an active role in their treatment. Its use could save time and resources for these professionals, improving the quality of the treatments.

## Figures and Tables

**Table 1:** Characteristics of studies.

Author, Year, Country	Study Sample (*n*)	Sample (% Men)	Sample Age (Year ± SD)	Etiology (%)	Extent of Disease (%)	Treatment Estatus (%)	Caregiver: Relation-ship (%)	Quality Assessment Downs and Black (Risk of Bias)
De Wit et al. [20] (1997), Netherlands	RCT:313 EG1: with district nurse (53) EG2: without district nurse (106) CG1: with district nurse (51) CG2: without district nurse (103)	37.4	55.5 ± 12.4	H&N: 18 (5.8) Digestive: 38 (12.1) Respiratory: 34 (10.9) Breast: 94 (30) Bone: 43 (13.7) Genitourinary: 74 (23.6) Others: 35 (11.2)	Local: 45 (14.4) Regional: 60 (19.2) Metastatic: 182 (58.1) Unknown: 9 (2.9) Not applicable: 17 (5.4)	Surgery: 50 (18) ChT: 112 (36) RT: 39 (12) HT: 21 (6) No treatment: 70 (22) Other/Unknow: 21 (7)	Informal caregiver	21 (Some concerns of bias)
Wells et al. [21] (2003), USA	RCT: 64 EG1: pain hot line (21) EG2: weekly calls (19) CG (24)	66	53 ± 14.5	H&N: 21(39)	No metastatic disease: 23(43) Local metastasis: 12(22) Distant metastasis: 18(35)	NR	Informal caregiver: Spouse (64) Adult child (14) Sibling (8)	20 (High risk of bias)
Miaskowski et al. [22] (2004), USA	RCT: 212 EG (93) CG (81)	51 EG: 31.2 CG: 27.2	EG: 60 ± 11.8 CG: 58.8 ± 12.9	Breast: 90 (52.02) Prostate: 22 (12.72) Respiratory: 22 (12.72) Other: 38 (22.54)	Metastatic: 173 (100)	ChT: 70 (40.22) RT: 29 (16.66) HT: 54 (31.03) Biotherapy: 2 (1.15) No treatment: 18 (10.64)	Informal caregiver	22 (High risk of bias)
Keefe et al. [23] (2005), USA	RCT: 78 EG: 41 CG: 37	56.1	60.49	NR	Metastatic: 78 (100)	Palliative care: 78 (100)	Informal caregivers: Spouses (54) daughters (14) unknown (32)	21 (Low risk of bias)
Lin et al. [24] (2006), Taiwan	RCT: 61 EG: 31 CG: 30	39.64 EG: 38.71 CG: 40	EG: 59.45 ± 16.90 CG: 55 ± 14.38	NR	Metastatic: 45 (73.77)	ChT: 9 (14.75) RT: 25 (40.98) ChT + RT: 4 (6.55) No treatment: 23 (37.70)	Informal caregiver	18 (High risk of bias)
Rustoen et al. [25] (2012), USA	RCT: 179 EG: 87 CG: 92	51.39 EG: 47.1 CG: 55.4	EG: 64.32 ± 11.4 CG: 67.38 ± 11.4	Breast: 66 (36.87) Prostate: 65 (36.31) Colon: 18 (10.06) Other: 30(16.76) Gynecological: 13 (33.3) Other: 13 (33.3)	Metastatic: 179 (100)	ChT: 62 (34.63) RT: 58 (32.40) HT: 48 (26.82) 15 (38.4) Other: 4 (10.4)	Informal caregiver	19 (Some concerns of bias)
Koller et al. [26] (2017), Germany	Pilot RCT: 39 EG: 20 CG: 19	51.1 EG: 40 CG: 63.8	56.6 ± 10.6	Gastrointes-tinal: 13 (33.3) Breast and gynecological: 13 (33.3) Other: 13 (33.3)	NR	Curative: 20 (51.3) Palliative: 15 (38.4) Other 4 (13.3)	Informal caregiver	21 (Some concerns of bias)

Abbreviations: CG, control group; ChT, chemotherapy; EG, experimental group; H&N, head and neck; HT, hormonal therapy; RCT, randomized control trial; RT, radiotherapy.

**Table 2:** Characteristics of the experimental intervention.

Author, (Year)	Experimental Intervention/ Components	Comparative Intervention	Face-to-Face vs. Distance Supervised vs. Non-Supervised Individual vs. Group	Total Sessions; Days/Week; Total Minutes	Outcomes	Main Results
De Wit et al. [20] (1997)	*Taxonomy* 1. Pain knowledge 2. Advising changing routines 3. Advising medication use 4. Barriers for pain management *Specific Pain Topic* Definition, causes, control (pharmaco-logical, analgesia and other techniques,), non-adherence, side effects, misconceptions, and pain levels recording.	Usual care	Mixed Supervised Individual	3; every 3 days; 15–60	Pain experience (MPQ-DLV) Present and average pain intensity (NRS) Pain knowledge (PKQ-DLV) QoL (EORTC QLQ-C30)	*p* < 0.05 for NPRS and PKQ-DLV in favor of EG1 and EG2 *p* > 0.05 for the rest of outcomes
Wells et al. [21] (2003)	*Taxonomy* 1. Pain knowledge 2. Advising medication use 3. Barriers for pain management *Specific Pain Topic* Control (pharmaco-logical, analgesia), non-adherence, side effects, and pain levels recording.	Usual care	Mixed Supervised Individual	EG1: 1, 1× Week; 15–45 EG2: 1, 1× Week; 15–45 EG3: 4, 1× Week; 15	Pain experience (BPI-SF) Barriers to pain (BQ-r) Family pain beliefs (FPQ) Medication Use (PMI-r)	*p* < 0.05 for FPQ in favor of EG *p* > 0.05 for the rest of outcomes
Miaskow-ski et al. [22] (2004)	*Taxonomy* 1. Pain knowledge 2. Advising medication use 3. Barriers for pain management *Specific Pain Topic* Reported needs, communic-ating skills, control (analgesia), side effects, and pain levels recording.	Usual care	Mixed Supervised Individual	6; 1× Week; NR	Least, average and worst pain intensity (NRS) Medication use	*p* < 0.05 for NRS and medication use in favor of EG
Keefe et al. [23] (2005)	*Taxonomy* 1. Pain knowledge 2. Advising changing routines 3. Barriers for pain management *Specific Pain Topic* Reported needs, control (pharmaco-logical, analgesia), side effects, coping strategies (pleasant imagery, activity pacing, activity-rest cycling), communica-ting skills, and relief barriers.	Usual care	Face-to-face Supervised Individual	3, biweekly; 45–60	Pain experience (BPI) QoL (FACT-G, V.4) Partner self-efficacy (CPSS) Caregiver strain (CSI) Partners’ mood (PMS-B)	*p* < 0.05 for CPSS and CSI *p* > 0.05 for the rest of outcomes
Lin et al. [24] (2006)	*Taxonomy* 1. Pain knowledge 2. Advising medication use 3. Barriers for pain management *Specific Pain Topic* Control (analgesia), side effects, and pain levels recording.	Usual care	Mixed Supervised Individual	3; weekly-biweekly; 30–40	Pain experience (BPI) Barriers to pain (BQT) Medication use (KPS)	*p* < 0.05 for BPI, BQT in favor of EG *p* > 0.05 for the rest of outcomes
Rustoen et al. [25] (2012)	*Taxonomy* 1. Pain knowledge 2. Advising changing routine *Specific Pain Topic* Reported needs, control (analgesia), side effects, communica-ting skills, and pain levels recording.	Usual care	Mixed Supervised Individual	6; weekly; NR	Pain experience (PES) Least, average and worst pain intensity (NRS) Medication use	(*p* < 0.001). *p* > 0.05 for PES *p* > 0.05 for the rest of outcomes
Koller et al. [26] (2017)	*Taxonomy* 1. Pain knowledge 2. Advising changing routines *Specific Pain Topic* Reported needs, misconcep-tions, control (analgesia), side effects, and communica-ting skills.	Usual care	Face-to-face Supervised Individual	1; NR; NR	Average and worst pain intensity (NRS) Knowledge pain (PPQ) Self-efficacy (PSQ) Barriers to cancer (BQ) QoL (MOS-SF)	*p* > 0.05 for PPQ *p* < 0.05 for the rest of outcomes

Abbreviations: API, average pain intensity; BQ, German version of the barriers questionnaire; BQ-r, barriers questionnaire revised; BQT, barriers questionnaire Taiwan form; BPI, brief pain index; BPI-SF, brief pain intensity short form; CPSS: Chronic Pain Self-efficacy Scale; CSI: Caregiver Strain Index; ECOG-PS, German version of the eastern cooperative oncology group; EORTC QLQ-C30, European organization for research and treatment of cancer core quality of life questionnaire; FPQ, family pain questionnaire; KPS: Karnofsky Performance Status; MOS-SF, medical outcome study short-form; MPQ-DLV, Dutch language version of the McGill pain questionnaire; NRS, numeric rating scale; PES, pain experience scale; PHQ, patient health questionnaire; PK: pain knowledge; PKQ-DLV, pain knowledge questionnaire; PMI-r, pain management index; PMS-B: Profile of Mood Stattes-B; PPI, present pain intensity; PSQ, self-efficacy questionnaire; WPI, worst pain intensity.

## Data Availability

No additional data are available.

## Abbreviations

Table 1: RCT, randomized control trial; EG, experimental group; CG, control group; H&N, head and neck; ChT, chemotherapy; RT, radiotherapy; HT, hormonal therapy. Table 2: MPQ-DLV, Dutch language version of the McGill pain questionnaire; PKQ-DLV, pain knowledge questionnaire; NRS, numeric rating scale; EORTC QLQ-C30: European organization for research and treatment of cancer core quality of life questionnaire; PPI, present pain intensity; API, average pain intensity; WPI, worst pain intensity; BPI-SF, brief pain inventory short form; BQ-r, barriers questionnaire-revised; FPQ, family pain questionnaire; PMI-r, pain management index; BQT, barriers questionnaire Taiwan form; PES, pain experience scale; PSQ, self-efficacy; BQ, German version of the barriers questionnaire; MOS-SF, medical outcome study short-form; ECOG-PS, German version of the eastern cooperative oncology group performance; PHQ, patient health questionnaire; BPI, brief pain inventory.
